# Vitamin D Metabolism Parameters and Cytokine Profile in COVID-19 Patients with Bolus Cholecalciferol Supplementation

**DOI:** 10.3390/diagnostics14131408

**Published:** 2024-07-02

**Authors:** Tatiana L. Karonova, Arina A. Mikhaylova, Ksenia A. Golovatyuk, Alena T. Chernikova, Zoia R. Korobova, Natalia E. Liubimova, Anna A. Starshinova, Dmitry A. Kudlay, Areg A. Totolian, Evgeny V. Shlyakhto

**Affiliations:** 1 Almazov National Medical Research Centre, 2, Akkuratov Str., St. Petersburg 197341, Russia; karonova@mail.ru (T.L.K.); armikhaylova@yandex.ru (A.A.M.); ksy2411@mail.ru (K.A.G.); arabicaa@gmail.com (A.T.C.); e.shlyakhto@almazovcentre.ru (E.V.S.); 2 Saint Petersburg Pasteur Institute, Saint-Petersburg 197101, Russia; zoia-korobova@mail.ru (Z.R.K.); natelu@mail.ru (N.E.L.); totolian@spbraaci.ru (A.A.T.); 3 Department of Pharmacognosy and Industrial Pharmacy, Faculty of Fundamental Medicine, Lomonosov Moscow State University, Moscow 119991, Russia; d624254@gmail.com; 4 Institute of Immunology, Moscow 115478, Russia

**Keywords:** COVID-19, vitamin D, 25(OH)D, 1,25(OH)2D, cholecalciferol, cytokines, inflammatory markers

## Abstract

Recent studies have demonstrated the relationship between vitamin D deficiency, infection severity and mortality from COVID-19. This study aimed to analyze the vitamin D metabolites and cytokine expression levels of COVID-19 patients who were hospitalized with bolus cholecalciferol supplementation. Materials and methods: This study represents the next stage of the open-label randomized pilot conducted by the Almazov National Medical Research Centre. A total of 44 hospitalized patients, comparable in demographic, clinical, laboratory and instrumental baseline characteristics, with moderate/severe COVID-19 were included. All patients had similar doses of concomitant corticosteroid therapy. Twenty-two patients received 50,000 IU cholecalciferol on the first and eighth days of hospitalization. The serum 25(OH)D, 1,25(OH)2D and 28 plasma cytokines were estimated for each group initially and on the ninth day of hospitalization. Results: Initially, there were no differences in the 1,25(OH)2D and cytokine levels in patients with vitamin D deficiency and normal 25(OH)D. Bolus cholecalciferol therapy at a total dose of 100,000 IU led to an increase in 25(OH)D levels in hospitalized patients with COVID-19, while the levels of the active metabolite (1,25(OH)2D) did not show significant differences between the groups or in its increased level over time, regardless of cholecalciferol supplementation. Furthermore, cholecalciferol supplementation at a total dose of 100,000 IU did not affect the majority of the cytokines estimated on the ninth day of hospitalization, except for the pro-inflammatory marker IL-1b, the concentration of which was lower in the group of patients without vitamin D supplementation. Conclusions: The 25(OH)D level was positively associated with an anti-inflammatory immune response, but cholecalciferol supplementation at a total dose of 100,000 IU did not affect the active-form vitamin D or cytokine expression levels. This fact may be explained by the impact of corticosteroid therapy, and it requires further investigation in a post-COVID-19 context.

## 1. Introduction

Investigations on the prevention and treatment of acute respiratory viral infections remain an essential task during the COVID-19 pandemic. Recent studies have demonstrated the relationship between vitamin D deficiency, infection severity and mortality from COVID-19 [1,2,3,4]. Some randomized intervention studies suggest that vitamin D supplementation may improve clinical and laboratory parameters, immunological markers and outcomes in patients affected by COVID-19 [5,6,7]. 

Taking into account the crucial impact of the T-cell-mediated immune response in SARS-CoV-2 infection [8], it is necessary to mention the role of vitamin D in the modulation of the adaptive immune system through its direct effects on T cell activation.

In particular, the biological effects of vitamin D are mediated through nuclear receptors (VDRs) that regulate multiple genes’ expression [9]. Consequently, the binding of 1,25(OH)2D to VDRs on T lymphocytes results in a reduction in pro-inflammatory cytokines and an increase in anti-inflammatory cytokine gene expression, indicating that vitamin D may impact the development of the cytokine storm linked to COVID-19 [10,11]. Biologically active 1,25(OH)2D is primarily produced through 1α-hydroxylation, predominantly in the kidneys [12]. However, 1-α-hydroxylase (CYP27B1) in macrophages and monocytes, when interacting with pathogens, can convert 25(OH)D to 1,25(OH)2D, leading to an increase in the production of cathelicidin LL-37 with antiviral properties [13,14,15]. Despite the significance of the active form of vitamin D, the assessment of serum 1,25(OH)2D in clinical practice is constrained due to its low concentration and short half-life, the labor-intensive methodology and the high cost [16].

It is crucial to ascertain whether cholecalciferol therapy influences clinical and laboratory parameters during the acute COVID-19, particularly given the mounting evidence of the significance of vitamin D deficiency in infectious diseases’ pathology [17]. 

Thus, the aim of this study was to evaluate the levels of 25(OH)D and 1,25(OH)2D and the cytokine expression levels at baseline and on the ninth day after bolus cholecalciferol supplementation in hospitalized patients with COVID-19. 

## 2. Materials and Methods

This study is one stage of the open-label randomized pilot conducted by the Almazov National Medical Research Centre [18]. The randomized single-center open-label study was performed from 30 November 2020 to 20 March 2021, when the Almazov National Medical Research Centre (St. Petersburg, Russia) was transformed into a dedicated hospital for COVID-19 patients.

The inclusion criteria were as follows: age from 18 to 75 years; diagnosis of moderate to severe COVID-19 confirmed by multislice computed tomography (MSCT) of the chest and/or real-time polymerase chain reaction (PCR). The exclusion criteria were as follows: diseases affecting vitamin D metabolism; estimated glomerular filtration rate less than 45 mL/min/1.73 m^2^; and daily intake of vitamin D supplements at a dose of more than 1000 IU. 

Regarding hospital outcomes, all patients were discharged from the hospital, with no recorded mortality. 

The patients’ baseline characteristics are presented in Table 1.

No participants were vaccinated as a general vaccination was not yet accessible at this time in the Russian Federation [19].

Forty-two patients, matched in terms of their demographic, clinical, instrumental and laboratory parameters (including vitamin D status), were selected from the two randomized groups of the study, which have been described previously [18]. In Group 1 (*n* = 22), 100,000 IU of water-soluble cholecalciferol (oral bolus) was added to the standard COVID-19 therapy; patients in Group 2 (*n* = 22) did not receive cholecalciferol supplementation. 

Blood samples for measurements were taken in the morning from the cubital vein and centrifuged, aliquoted and stored in a freezer at a temperature of −70 °C before the laboratory testing.

The serum 25(OH)D level was assessed on the 1st and 9th days of hospitalization with a biochemical analyzer, the Architect c8000 Processing Module (Abbott Laboratories, Chicago, IL, USA), using calibrators and control serums from the manufacturer—reference range, 3.4–155.9 ng/mL. The serum 1,25(OH)2D level was determined on the 1st and 9th days of hospitalization by an enzyme immunoassay using a commercial kit (ELISA Kit for 1,25-dihydroxyvitamin D3 (DHVD3); CEA467Ge; detection range: 24.69–2000 pg/mL; Cloud-Clone Corp., Wuhan, China). 

The assessment of the plasma cytokine profile (TNF-α, IFN-γ, GM-CSF, IL-1b, IL-2, IL-4, IL-6, IL-8, IL-10, IL-12(p70), IL-17A, IL-21) was performed using the MAGPIX System multiplex fluorescent analyzer with Milliplex MAP Kit calibrators for each group, initially and on the 9th day of hospitalization. The unit of measurement for all cytokines was picograms per milliliter (pg/mL). 

COVID-19 medications, including the total dose of glucocorticosteroids (GCS) administered by the 9th day of hospitalization, were analyzed in all patients. The calculation of the dexamethasone-equivalent dose of GCS was conducted using the Steroid Conversion Calculator software [20].

Statistical analysis was performed using Jamovi, v. 2.3.2, 2022 (Jamovi Project, Sydney, Australia). Data are presented as the median and interquartile range (25th; 75th percentiles): Me [Q25; Q75]. The Mann–Whitney U-test was used to assess the statistical significance of the differences between two independent groups. The Wilcoxon W-test was used to assess the statistical significance of the differences between two dependent indicators. Spearman’s correlation test was used for correlation analysis. Statistical significance was defined as *p* < 0.05.

## 3. Results

Most patients from both groups had vitamin D deficiency or insufficiency at baseline (Table 2). 

**Figure 1:**
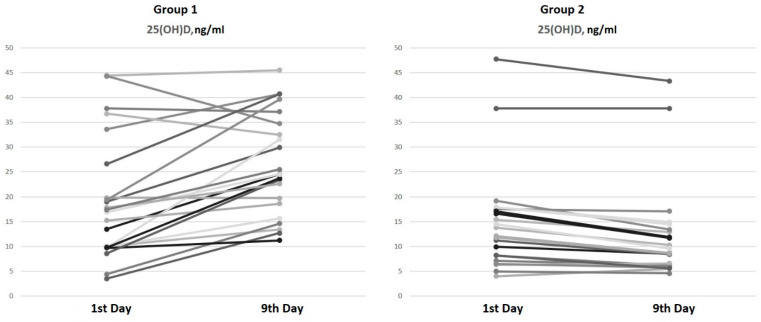
Serum 25(OH)D dynamics from the 1st to the 9th day of hospitalization.

On the ninth day of hospitalization, an increase in serum 25(OH)D levels of 45.8% [16.9; 98.4] in patients from Group 1 (bolus cholecalciferol therapy) and a decrease in 25(OH)D levels of 17.9% in patients from Group 2 (without cholecalciferol therapy) were observed (Figure 1). 

**Figure 2:**
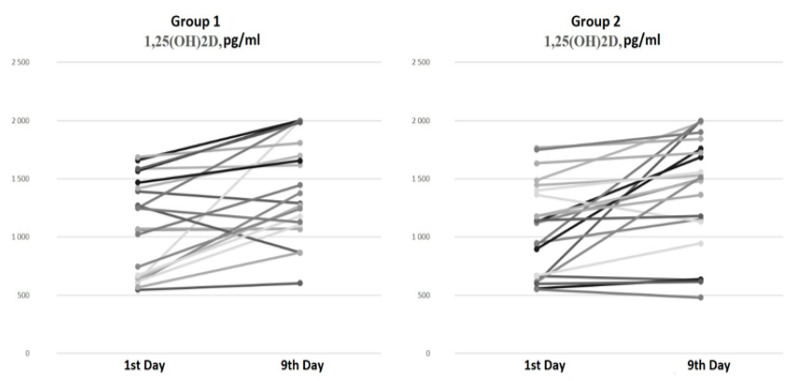
Serum 1,25(OH)2D dynamics from the 1st to the 9th day of hospitalization.

There were no differences in the serum 1,25(OH)2D levels at baseline and on the ninth day of hospitalization between the groups (Figure 2). 

The paired comparison analysis on the first and ninth days of hospitalization revealed an increase in both the 25(OH)D (*p* < 0.001) and 1,25(OH)2D (*p* < 0.001) levels by the ninth day in Group 1 (bolus cholecalciferol therapy). Despite the negative dynamics of the 25(OH)D level by the ninth day (*p* < 0.001) in Group 2 (without cholecalciferol therapy), an increase in the 1,25(OH)2D (*p* < 0.001) concentration was also observed.

The correlation analysis between the active-form vitamin D levels and clinical parameters revealed a significant positive association between the 1,25(OH)2D levels on the ninth day of hospitalization and the number of hospitalization days (r = 0.38, *p* = 0.011).

Most patients (95.5%) from both groups received GCS during hospitalization (Table 3). The total GCS dose equivalent to dexamethasone by the ninth day did not differ between the groups (*p* = 0.32). One patient in Group 2 took GCS for rheumatoid arthritis before hospitalization.

Subsequently, an assessment of the dynamics of the pro-/anti-inflammatory cytokine expression levels was conducted (Table 4). 

As previously outlined (Table 3), the patients in both groups received GCS therapy in equivalent doses, with an equivalent number of patients receiving pathogenetic therapy with IL-6 receptor antagonists (*n* = 7 in each group). Considering the known impact of the agonist IL-6 receptor on the cytokine profile, the cytokine levels were evaluated on the ninth day of hospitalization, excluding these patients (*n* = 30). 

It was observed that the duration from the onset of the initial COVID-19 symptoms to the first cytokine control assessment and the initiation of therapy did not differ significantly between the two groups, averaging 7 [3; 10] days in Group 1a and 8 [6; 9] days in Group 2a (*p* = 0.44). 

**Figure 3:**
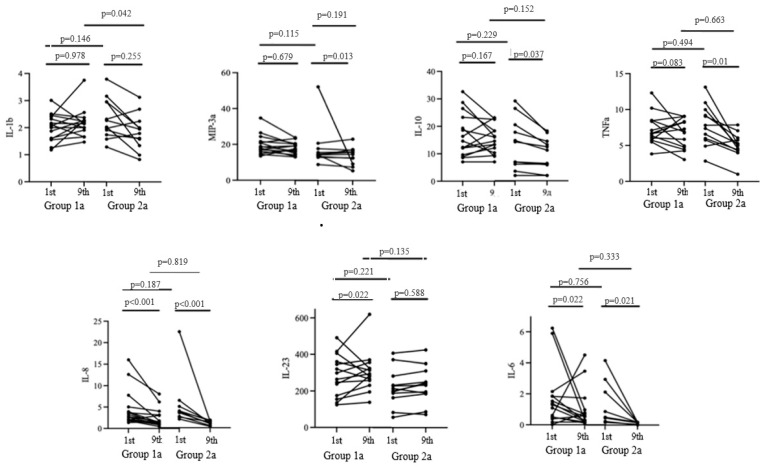
Cytokine profile dynamics from the 1st to the 9th day of the hospitalization.

The duration from the onset of the initial COVID-19 symptoms to the cytokine assessment during cholecalciferol therapy was 15 [11; 18] days in Group 1a and 16 [14; 18] days in Group 2a (*p* = 0.48) (Figure 3).

The baseline expression levels of pro-inflammatory and anti-inflammatory cytokines were comparable in both groups. On the ninth day of hospitalization, a significant decrease in the concentrations of IL-10, MIP-3a, and TNFa was observed in Group 2a, while an increase in the level of IL-23 was noted in Group 1a (*p* < 0.05). 

Both groups exhibited a decrease in IL-6 and IL-8 levels. However, no significant differences in the aforementioned parameters were observed by the ninth day of hospitalization in the study groups. Meanwhile, significant differences in the level of IL-1b were observed on the ninth day of hospitalization, with a level of 2.22 [1.96; 2.41] in Group 1a and 1.79 [1.58; 2.0] in Group 2a.

Thus, cholecalciferol supplementation at a dose of 100,000 IU did not affect the majority of the cytokines estimated on the ninth day of hospitalization, except for the pro-inflammatory marker IL-1b, the concentration of which was lower in the group of patients without vitamin D supplementation.

## 4. Discussion

There have been limited studies focusing on vitamin D metabolism during the acute period of COVID-19; for example, Povaliaeva A. and colleagues observed elevated serum levels of 1,25(OH)2D in hospitalized patients with acute COVID-19 compared to the control group. They also noted a decrease in these levels during the long-term follow-up period. The authors proposed that the increase in extrarenal 1α-hydroxylase activity in the acute phase of COVID-19 may contribute to the rise in 1,25(OH)2D levels [21].

Furthermore, the use of GCS therapy in patients with moderate and severe COVID-19 may also impact vitamin D metabolism. The findings from the NHANES cross-sectional study revealed a link between GCS therapy and vitamin D deficiency. It is widely known that GCS reduces the serum 25(OH)D concentration by upregulating 1α-hydroxylase expression, but there are no data about the effects on the levels of 1,25(OH)2D [22,23]. However, the level of 1,25(OH)2D does not necessarily reflect vitamin D sufficiency, unlike its inactive precursor, 25(OH)D. Several studies have shown that vitamin D supplementation increases the levels of 25(OH)D without affecting its active form [23,24,25,26].

Our findings align with the previously reported data. Bolus cholecalciferol therapy at a total dose of 100,000 IU led to an increase in 25(OH)D levels in hospitalized patients with COVID-19, whereas COVID-19 therapy without cholecalciferol supplementation was associated with a decrease in 25(OH)D levels. Interestingly, the levels of the active metabolite (1,25(OH)2D) did not show significant differences between the groups and increased over time regardless of cholecalciferol supplementation. These characteristics of vitamin D metabolism during the acute phase of COVID-19 may stem from alterations in 1α-hydroxylase activity due to both the active infectious process and GCS therapy, leading to an increase in the 1,25(OH)2D concentration independently of the basal vitamin D status.

After excluding patients who had received IL-6 receptor antagonist drugs during their hospitalization, we evaluated alterations in the cytokine profile following the administration of bolus cholecalciferol therapy. The analysis revealed that cholecalciferol supplementation at a total dose of 100,000 IU did not have a significant impact on the majority of the cytokines examined on the ninth day of hospitalization, with the exception of the pro-inflammatory marker IL-1b, which exhibited lower levels in patients not undergoing vitamin D therapy.

Previous studies investigating cytokine expression levels have yielded conflicting results [27,28,29,30]. The authors attribute these divergent trends to variations in the timing of the cytokine assessments throughout the course of the illness, differing dosages of vitamin D supplementation, and, primarily, the concomitant use of GCS therapy. Regarding the time points of the cytokine profile evaluations, the relatively lengthy interval between the onset of acute respiratory illness symptoms and the commencement of vitamin D administration (median 7–8 days), alongside the necessary duration for the active form of vitamin D to modulate the anticipated immune response, may have mitigated its impact on the clinical and biochemical outcomes.

Furthermore, a recent study by Liu C. et al. demonstrated that critically ill COVID-19 patients experience a peak in inflammation, marked by the second wave of the “cytokine storm”, approximately 17–23 days after symptom onset [31]. In this study, the median duration until the assessment of the second data point was 15–16 days. It is plausible that the evaluation was premature in identifying shifts in the cytokine profile. Additionally, all patients received GCS therapy equating to a total dose of 120–146 mg of dexamethasone by the ninth day of hospitalization; this serves as the principal pathogenetic and immunosuppressive therapy for COVID-19 and predominantly influences the cytokine response. According to the literature data, glucocorticoids suppress the production and activity of various cytokines, effectively dampening both the initial immune response and the subsequent immune regulation. Specifically, they significantly reduce the levels of early-phase cytokines, responsible for initiating the inflammatory cascade (IL-1 beta and TNF-alpha), and immunomodulatory cytokines (IL-2, IL-3, IL-4, IL-5, IL-10, IL-12, IFN-gamma, IL-6, IL-8, GM-CSF), crucial in orchestrating the immune response. Interestingly, even the production of the anti-inflammatory cytokine IL-10, which typically helps to resolve inflammation, is also suppressed by glucocorticoids [32,33].

To summarize, despite the positive impact of vitamin D treatment on the course of COVID-19 and patients’ laboratory parameters demonstrated in a recently published paper [18], we did not observe a significant effect on 1,25(OH)2D or the cytokine profile. This finding could be explained by the influence of concomitant therapy and warrants further investigation.

## 5. Study Limitations

This study did not include patients with mild COVID-19 infection. All patients in this study received GCS therapy, and there was no comparison group without GCS therapy. There is a lack of data on 24,25(OH)2D and other vitamin D metabolite concentrations. Mass spectrometry methods were not used for the metabolite assessment.

## 6. Conclusions

Regardless of cholecalciferol therapy, all patients showed an increase in the level of 1,25(OH)2D, which may indicate vitamin-D-associated immunomodulatory properties in patients with COVID-19. 

At the same time, despite the positive impact of vitamin D treatment on the course of COVID-19 and patients’ laboratory parameters demonstrated in a recently published paper [18], we did not observe a significant effect on 1,25(OH)2D or the cytokine profile. This finding could be explained by the influence of concomitant therapy and warrants further investigation, alongside interventional studies in a post-COVID-19 context.

## Figures and Tables

**Table 1:** Baseline characteristics of hospitalized COVID-19 patients.

Parameter	Group 1 (*n* = 22)	Group 2 (*n* = 22)	*p*
Age, years, Me + IQR [25; 75]	58.5 [55.5; 66.8]	65 [56.3; 70]	0.39
Gender, females, *n* (%)	11 (50)	12 (54.5)	0.78
BMI, kg/m^2^, Me + IQR [25; 75]	28.6 [24.7; 33.2]	29.5 [27.2; 33.1]	0.5
Admission day (from the first manifestation), Me + IQR [25; 75]	7 [3; 10]	8 [6; 9]	0.44
Lung involvement, %, Me + IQR [25; 75]	35.5 [20; 45.8]	37.5 [16.3; 49.5]	0.76
Days of hospitalization, Me + IQR [25; 75]	16 [12; 20]	15 [14; 22]	0.86
C-reactive protein (1st day), mg/L	28.7 [19.4; 31.9]	36.7 [17.5; 83.8]	0.23
C-reactive protein (9th day), mg/L	3.0 [0.7; 7.7]	5.6 [1.3; 14.9]	0.11

Data are presented as median and interquartile range—Me [Q25; Q75], absolute and relative frequencies—*n* (%), BMI—body mass index.

**Table 2:** Vitamin D metabolite levels on the 1st and 9th days of hospitalization.

Parameter	Group 1 (*n* = 22)	Group 2 (*n* = 22)	*p*
25(OH)D (1st day), ng/mL, Me + IQR [25; 75]	17.1 [9.83; 24.9]	12.9 [8.2; 17.4]	0.19
25(OH)D (1st day), *n* (%)			0.13
Normal	5 (22.7)	2 (9)	
Insufficiency	1 (4.5)	0 (0)	
Deficiency	16 (72.7)	20 (91)	
25(OH)D, ng/mL (9th day), Me + IQR [25; 75]	24.5 [18.9; 34.2]	9.15 [6.38; 13.3]	<0.001
25(OH)D, ng/mL (9th day), *n* (%)			<0.001
Normal	8 (36.4)	2 (9)	
Insufficiency	7 (31.8)	0 (0)	
Deficiency	7 (31.8)	20 (91)	
Δ25(OH)D, %	45.8 [16.9; 98.4]	−17.9 [−27.9; 0]	<0.001
1,25(OH)2D (1st day), pg/mL, Me + IQR [25; 75]	1158 [649; 1455]	1127 [665; 1390]	0.71
1,25(OH)2D (9th day), pg/mL, Me + IQR [25; 75]	1333 [1111; 1779]	1506 [1137; 1750]	0.96
Δ1,25(OH)2 D, %	18.6 [3.12; 39.3]	13.0 [4.48; 28.3]	0.74

Data are presented as median and interquartile range—Me [Q25; Q75], absolute and relative frequencies—*n* (%); 25(OH)D—25-hydroxycholecalciferol, 1,25(OH)2D—1,25-dihydroxycholecalciferol.

**Table 3:** Characteristics of glucocorticosteroid therapy.

Parameter	Group 1 (*n* = 22)	Group 2 (*n* = 22)	*p*
GCS therapy before admission, *n* (%)	0 (0)	1 (4.5)	0.34
GCS therapy during hospitalization, *n* (%)	21 (95.5)	21 (95.5)	1.0
Dexamethasone therapy, *n* (%)	18 (81.8)	19 (86.4)	0.7
Prednisolone therapy, *n* (%)	6 (27.2)	2 (9)	0.13
Methylprednisolone therapy, *n* (%)	3 (13.6)	1 (4.5)	0.31
Total GCS dose (equivalent to dexamethasone) by the 9th day of hospitalization, mg, Me + IQR [25; 75]	120 [45; 152]	146 [78; 195]	0.32

Note. Data are presented as median and interquartile range—Me [Q25; Q75], absolute and relative frequencies—*n* (%); GCS—glucocorticosteroids.

**Table 4:** Cytokine profile dynamics from the 1st and to the 9th day of hospitalization.

Parameter	Group 1a (*n* = 16)		*p*_1_	Group 2a (*n* = 15)		*p*_2_	p*	*p* ^#^
	1st Day	9th Day		1st Day	9th Day			
TNFα, pg/mL	6.71 [6.20; 9.64]	5.86 [4.85; 08.07]	0.08	7.11 [5.48; 8.49]	5.79 [4.29; 7.09]	0.01	0.49	0.66
IFNγ, pg/mL	65.1 [45.0; 77.2]	54.9 [45.0; 74.0]	0.56	56.4 [48.1; 80.7]	62.2 [44.3; 73.3]	0.54	0.76	0.87
GM-CSF, pg/mL	15.7 [14.2; 18.6]	17.0 [15.5; 20.6]	0.12	13.5 [9.45; 24.6]	18.4 [14.4; 21.5]	0.17	0.59	0.95
IL-1b, pg/mL	2.27 [1.92; 2.70]	2.22 [1.96; 2.41]	0.98	2.00 [ 1.58; 2.32]	1.79 [1.58; 2.00]	0.26	0.15	0.04
IL-2, pg/mL	2.73 [2.28; 3.41]	3.04 [2.56; 3.64]	0.30	2.13 [1.77; 2.94]	2.48 [1.82; 03.04]	0.46	0.29	0.18
IL-4, pg/mL	26.0 [16.3; 39.9]	26.7 [23.5; 34.3]	0.23	16.3 [11.4; 26.3]	17.0 [13.5; 28.8]	0.66	0.25	0.09
IL-5, pg/mL	2.70 [2.22; 3.73]	3.34 [2.16; 4.10]	0.39	3.13 [02.06; 3.39]	2.65 [2.44; 3.44]	0.97	0.71	0.52
IL-6, pg/mL	0.880 [0.482; 2.01]	0.231 [0.183; 0.741]	0.02	1.3 [0.554; 3.49]	0.207 [0.183; 1.07]	0.02	0.76	0.33
IL-8, pg/mL	3.70 [2.56; 6.40]	1.43 [0.901; 1.70]	<0.001	3.22 [2.57; 3.73]	1.05 [1.02; 1.61]	<0.001	0.19	0.82
IL-10, pg/mL	17.6 [10.9; 24.8]	14.4 [11.6; 18.2]	0.17	12.4 [6.81; 19.3]	10.1 [6.04; 13.4]	0.04	0.23	0.15
IL-12 (p70), pg/mL	2.07 [1.72; 3.14]	2.52 [2.02; 3.07]	0.33	1.92 [1.57; 3.16]	2.07 [1.21; 3.59]	0.61	0.72	0.71
IL-17A, pg/mL	7.46 [5.61; 9.37]	7.80 [7.25; 10.3]	0.12	8.76 [5.41; 12.2]	9.44 [6.43; 12.2]	0.79	0.79	0.74
IL-21, pg/mL	4.11 [3.48; 4.83]	3.92 [3.53; 4.64]	0.85	3.92 [3.63; 5.54]	3.92 [3.63; 5.26]	0.37	0.63	0.90
IL-23, pg/mL	261 [186; 361]	294 [188; 363]	0.02	211 [175; 320]	258 [221; 284]	0.59	0.22	0.14
MIP-3a, pg/mL	17.8 [15.3; 22.7]	16.9 [15.0; 19.8]	0.68	17.0 [14.0; 18.9]	14.9 [12.7; 17.2]	0.01	0.12	0.19

Note. *p*_1_—the significance of the differences in the cytokine levels on the 1st and 9th days of hospitalization in patients in Group 1a, *p*_2_—the significance of the differences in the cytokine levels on the 1st and 9th days of hospitalization in patients in Group 2a, p*—the significance of the differences in the cytokine levels between patients in Group 1a and Group 2a on the 1st day of hospitalization, *p*^#^—the significance of the differences in the cytokine levels between patients in Group 1a and Group 2a on the 9th day of hospitalization.

## Data Availability

The data generated and analyzed during this study are included in this published article. Additional information is available from the corresponding author on reasonable request.

## References

[1] Kaufman H.W., Niles J.K., Kroll M.H., Bi C., Holick M.F. (2020). SARS-CoV-2 positivity rates associated with circulating 25-hydroxyvitamin D levels. PLoS ONE.

[2] Karonova T.L., Golovatyuk K.A., Aquino A.D., Kalinina O.V., Chernikova A.T., Zaikova E.K., Lebedev D.A., Bykova E.S., Golovkin A.S. (2022). Vitamin D Status and Immune Response in Hospitalized Patients with Moderate and Severe COVID-19. Pharmaceuticals.

[3] Kaya M.O., Pamukçu E., Yakar B. (2021). The role of vitamin D deficiency on COVID-19: A systematic review and meta-analysis of observational studies. Epidemiol. Health.

[4] Karonova T.L., Andreeva A.T., Golovatyuk K.A., Bykova E.S., Skibo I.I., Grineva E.N., Shlyakhto E.V. (2021). SARS-CoV-2 morbidity depending on vitamin D status. Probl. Endocrinol..

[5] Zaazouee M.S., Abdalalaziz A.M., Elhady M.M., Ali O.A., Abdelbari T.M., Hasan S.M., Almadhoon H.W., Ahmed A.Y., Fassad A.S., Elgendy R. (2022). Hospital and laboratory outcomes of patients with COVID-19 who received vitamin D supplementation: A systematic review and meta-analysis of randomized controlled trials. Naunyn-Schmiedeberg’s Arch. Pharmacol..

[6] Ling S.F., Broad E., Murphy R., Pappachan J.M., Pardesi-Newton S., Kong M.F., Jude E.B. (2020). High-Dose Cholecalciferol Booster Therapy is Associated with a Reduced Risk of Mortality in Patients with COVID-19: A Cross-Sectional Multi-Centre Observational Study. Nutrients.

[7] Annweiler G., Corvaisier M., Gautier J., Dubée V., Legrand E., Sacco G., Annweiler C. (2020). Vitamin D Supplementation Associated to Better Survival in Hospitalized Frail Elderly COVID-19 Patients: The GERIA-COVID Quasi-Experimental Study. Nutrients.

[8] Kudlay D., Kofiadi I., Khaitov M. (2022). Peculiarities of the T Cell Immune Response in COVID-19. Vaccines.

[9] Pike J.W., Meyer M.B., Lee S.-M., Onal M., Benkusky N.A. (2017). The vitamin D receptor: Contemporary genomic approaches reveal new basic and translational insights. J. Clin. Investig..

[10] Cantorna M.T., Snyder L., Lin Y.-D., Yang L. (2015). Vitamin D and 1,25(OH)_2_D Regulation of T cells. Nutrients.

[11] van Etten E., Mathieu C. (2005). Immunoregulation by 1,25-dihydroxyvitamin D3: Basic concepts. J. Steroid Biochem. Mol. Biol..

[12] Prietl B., Treiber G., Pieber T.R., Amrein K. (2013). Vitamin D and Immune Function. Nutrients.

[13] Telcian A.G., Zdrenghea M.T., Edwards M.R., Laza-Stanca V., Mallia P., Johnston S.L., Stanciu L.A. (2017). Vitamin D increases the antiviral activity of bronchial epithelial cells in vitro. Antivir. Res..

[14] Kreutz M., Andreesen R., Krause S.W., Szabo A., Ritz E., Reichel H. (1993). 1,25-dihydroxyvitamin D3 production and vitamin D3 receptor expression are developmentally regulated during differentiation of human monocytes into macrophages. Blood.

[15] Barlow P.G., Svoboda P., Mackellar A., Nash A.A., York I.A., Pohl J., Davidson D.J., Donis R.O. (2011). Antiviral activity and increased host defense against influenza infection elicited by the human cathelicidin LL-37. PLoS ONE.

[16] Zelzer S., Goessler W., Herrmann M. (2018). Measurement of vitamin D metabolites by mass spectrometry, an analytical challenge. J. Lab. Precis. Med..

[17] Kozlov V.A., Tikhonova E.P., Savchenko A.A., Kudryavtsev I.V., Andronova N.V., Anisimova E.N., Golovkin A.S., Demina D.V., Zdzitovetsky D.E., Kalinina Y.S. (2021). Clinical Immunology—A Practical Guide for Infectious Disease Specialists.

[18] Karonova T.L., Golovatyuk K.A., Kudryavtsev I.V., Chernikova A.T., Mikhaylova A.A., Aquino A.D., Lagutina D.I., Zaikova E.K., Kalinina O.V., Golovkin A.S. (2022). Effect of Cholecalciferol Supplementation on the Clinical Features and Inflammatory Markers in Hospitalized COVID-19 Patients: A Randomized, Open-Label, Single-Center Study. Nutrients.

[19] Kudlay D., Svistunov A., Satyshev O. (2022). COVID-19 Vaccines: An Updated Overview of Different Platforms. Bioengineering.

[20] Steroid Conversion Calculator. https://www.mdcalc.com/calc/2040/steroid-conversion-calculator.

[21] Povaliaeva A., Bogdanov V., Pigarova E., Dzeranova L., Katamadze N., Malysheva N., Ioutsi V., Nikankina L., Rozhinskaya L., Mokrysheva N. (2022). Impaired Vitamin D Metabolism in Hospitalized COVID-19 Patients. Pharmaceuticals.

[22] Skversky A.L., Kumar J., Abramowitz M.K., Kaskel F.J., Melamed M.L. (2011). Association of glucocorticoid use and low 25-hydroxyvitamin D levels: Results from the National Health and Nutrition Examination Survey (NHANES): 2001–2006. J. Clin. Endocrinol. Metab..

[23] Dhawan P., Christakos S. (2010). Novel regulation of 25-hydroxyvitamin D_3_ 24-hydroxylase (24(OH)ase) transcription by glucocorticoids: Cooperative effects of the glucocorticoid receptor, C/EBPβ, and the Vitamin D receptor in 24(OH)ase transcription. J. Cell. Biochem..

[24] Akeno N., Matsunuma A., Maeda T., Kawane T., Horiuchi N. (2000). Regulation of vitamin D-1alpha-hydroxylase and -24-hydroxylase expression by dexamethasone in mouse kidney. J. Endocrinol..

[25] Chapuy M.C., Chapuy P., Meunier P.J. (1987). Calcium and vitamin D supplements: Effects on calcium metabolism in elderly people. Am. J. Clin. Nutr..

[26] Barger-Lux M.J., Heaney R.P., Dowell S., Chen T.C., Holick M.F. (1998). Vitamin D and its Major Metabolites: Serum Levels after Graded Oral Dosing in Healthy Men. Osteoporos. Int..

[27] Seamans K.M., Cashman K.D. (2009). Existing and potentially novel functional markers of vitamin D status: A systematic review. Am. J. Clin. Nutr..

[28] Torres M., Casado G., Vigón L., Rodríguez-Mora S., Mateos E., Ramos-Martín F., López-Wolf D., Sanz-Moreno J., Ryan-Murua P., Taboada-Martínez M.L. (2022). Changes in the immune response against SARS-CoV-2 in individuals with severe COVID-19 treated with high dose of vitamin D. Biomed Pharmacother..

[29] Sharif-Askari F.S., Hafezi S., Sharif-Askari N.S., Alsayed H.A.H., Mdkhana B., Selvakumar B., Temsah M.-H., Saddik B., Al Anouti F., Halwani R. (2022). Vitamin D modulates systemic inflammation in patients with severe COVID-19. Life Sci..

[30] Fernandes A.L., Murai I.H., Reis B.Z., Sales L.P., Santos M.D., Pinto A.J., Goessler K.F., Duran C.S.C., Silva C.B.R., Franco A.S. (2023). Effect of a single high dose of vitamin D3 on cytokines, chemokines, and growth factor in patients with moderate to severe COVID-19. Am. J. Clin. Nutr..

[31] Liu C., Martins A.J., Lau W.W., Rachmaninoff N., Chen J., Imberti L., Mostaghimi D., Fink D.L., Burbelo P.D., Dobbs K. (2021). Time-resolved systems immunology reveals a late juncture linked to fatal COVID-19. Cell.

[32] Brattsand R., Linden M. (1996). Cytokine modulation by glucocorticoids: Mechanisms and actions in cellular studies. Aliment. Pharmacol. Ther..

[33] Beck I.M., Van Crombruggen K., Holtappels G., Daubeuf F., Frossard N., Bachert C., De Bosscher K. (2015). Differential cytokine profiles upon comparing selective versus classic glucocorticoid receptor modulation in human peripheral blood mononuclear cells and inferior turbinate tissue. PLoS ONE.

